# QTL associated with gummy stem blight resistance in watermelon

**DOI:** 10.1007/s00122-020-03715-9

**Published:** 2020-11-01

**Authors:** Winnie Gimode, Kan Bao, Zhangjun Fei, Cecilia McGregor

**Affiliations:** 1 Institute for Plant Breeding, Genetics & Genomics, University of Georgia, 1111 Plant Sciences Bldg, Athens, GA 30602 USA; 2 Boyce Thompson Institute for Plant Research, Cornell University, Ithaca, NY 14853 USA; 3 Department of Horticulture and Institute for Plant Breeding, Genetics & Genomics, University of Georgia, 1111 Plant Sciences Bldg, Athens, GA 30602 USA

## Abstract

**Key message:**

We identified QTLs associated with gummy stem blight resistance in an interspecific F_2:3_ *Citrullus* population and developed marker assays for selection of the loci in watermelon.

**Abstract:**

Gummy stem blight (GSB), caused by three *Stagonosporopsis* spp., is a devastating fungal disease of watermelon (*Citrullus lanatus*) and other cucurbits that can lead to severe yield losses. Currently, no commercial cultivars with genetic resistance to GSB in the field have been reported. Utilizing GSB-resistant cultivars would reduce yield losses, decrease the high cost of disease control, and diminish hazards resulting from frequent fungicide application. The objective of this study was to identify quantitative trait loci (QTLs) associated with GSB resistance in an F_2:3_ interspecific *Citrullus* mapping population (*N* = 178), derived from a cross between Crimson Sweet (*C. lanatus*) and GSB-resistant PI 482276 (*C. amarus*). The population was phenotyped by inoculating seedlings with *Stagonosporopsis citrulli* 12178A in the greenhouse in two separate experiments, each with three replications. We identified three QTLs (*ClGSB3.1*, *ClGSB5.1* and *ClGSB7.1*) associated with GSB resistance, explaining between 6.4 and 21.1% of the phenotypic variation. The genes underlying *ClGSB5.1* includes an NBS-LRR gene (*ClCG05G019540*) previously identified as a candidate gene for GSB resistance in watermelon*.* Locus *ClGSB7.1* accounted for the highest phenotypic variation and harbors twenty-two candidate genes associated with disease resistance. Among them is *ClCG07G013230*, encoding an Avr9/Cf-9 rapidly elicited disease resistance protein, which contains a non-synonymous point mutation in the DUF761 domain that was significantly associated with GSB resistance. High throughput markers were developed for selection of *ClGSB5.1* and *ClGSB7.1.* Our findings will facilitate the use of molecular markers for efficient introgression of the resistance loci and development of GSB-resistant watermelon cultivars.

**Electronic supplementary material:**

The online version of this article (10.1007/s00122-020-03715-9) contains supplementary material, which is available to authorised users.

## Introduction

Gummy stem blight (GSB) is a devastating fungal disease affecting cultivation of cucurbitaceous vegetable crops worldwide, leading to severe yield losses (Sherbakoff 1917; Chiu and Walker 1949; Sherf and MacNab 1986; Keinath 2011; Stewart et al. 2015). It has been reported to infect at least 12 genera and 23 species of Cucurbitaceae, including watermelon (*Citrullus lanatus*), cucumber (*Cucumis sativus*), cantaloupe and muskmelon (*Cucumis melo*), squash (*Cucurbita pepo*), and several different genera of gourds (Keinath 2011). The occurrence of GSB is intensified by warm and humid environments that are conducive for germination of the spores and disease development (Keinath et al. 1995; Robinson and Decker-Walters 1997; Keinath 2011; Babu et al. 2015; Stewart et al. 2015). GSB was formerly thought to be caused by a single pathogen: *Didymella bryoniae* (syn. *Stagonosporopsis cucurbitacearum*) (Aveskamp et al. 2010), but it has since been established that the disease is caused by three *Stagonosporopsis* species: *S. cucurbitacearum* (syn. *D bryoniae*), *S. citrulli*, and *S. caricae* (Stewart et al. 2015). Although morphologically similar, the three *Stagonosporopsis* species can be distinguished using polymerase chain reaction-based microsatellite markers (Brewer et al. 2015).

Current management of GSB in watermelon includes cultural practices and fungicide application. Due to the limited effectiveness of cultural practices on their own, fungicides remain critical for successful management of GSB (Stevenson et al. 2004; Keinath 2012). However, recent reports of differential fungicide resistance among the three causal *Stagonosporopsis* species presents a significant challenge to growers since the species cannot be differentiated based on symptoms (Brewer et al. 2015; Li et al. 2016, 2019; Newark et al. 2019). In addition, fungicide applications greatly increase production costs and their repeated use may have a negative impact on the environment, particularly if residues persist in the soil. The best alternative would be to utilize GSB-resistant cultivars, but currently commercial watermelon cultivars with high levels of genetic resistance to GSB have not been developed.

Due to the narrow genetic base of cultivated watermelon following domestication (Guo et al. 2013; Levi et al. 2017), the *Citrullus amarus*, a wild relative of watermelon (*C. lanatus*) (Chomicki and Renner 2015; Renner et al. 2017) has been a major source of disease resistance alleles in watermelon breeding (Boyhan et al. 1994; Guner 2005; Thies and Levi 2007; Tetteh et al. 2010; McGregor 2012; Wechter et al. 2012; Levi et al. 2017; Branham et al. 2019a, b). *Citrullus* germplasm sources with various levels of host resistance against GSB have been described (Sowell and Pointer 1962; Sowell 1975; Norton 1979; Gusmini et al. 2005). GSB resistance in *C. amarus* was described as early as 1962 in PI 189225 (Sowell and Pointer 1962) and later in PI 271778 (Sowell 1975; Norton 1979). Efforts to introgress resistance from these two sources into commercial cultivars was attempted, and led to the release of AU-Producer, AU-Jubilant, AU-Golden Producer and AU-Sweet Scarlet (Norton et al. 1986, 1993, 1995). However, these cultivars did not prove to be resistant in commercial production fields (Song et al. 2002). New sources of resistance that included accessions from both *C. amarus* and *C. lanatus* species were later described by Gusmini et al. (2005) and included PI 164248, PI 244019, PI 254744, PI 271771, PI 279461, PI 296332, PI 482379, PI 490383, PI 526233 and PI 482276. PI 482276 was found to be resistant to various isolates from all three *Stagonosporopsis* species (Gimode et al. 2019).

Initial studies of GSB resistance in PI 189225 reported that resistance was mediated by a single gene, *db* (Norton 1979). However, later studies on PI 189225, PI 482283, and PI 526233 found that many genes with minor effects are most likely responsible for this trait (Gusmini et al. 2017; Hassan et al. 2019; Ren et al. 2019). Recently, a quantitative trait locus (QTL) underlying GSB resistance in PI 189225 was described on chromosome 8 of watermelon (Ren et al. 2019). This QTL explains ~ 32% of the phenotypic variance in the population. Identification of loci linked to GSB resistance will facilitate development of molecular markers that would increase the efficiency of introgression of resistance loci into commercial watermelon cultivars.

The goal of the current study was to identify QTLs associated with GSB resistance in an F_2:3_ interspecific *Citrullus* population derived from a cross between Crimson Sweet and PI 482276 (Gusmini et al. 2005; Gimode et al. 2019), and to develop high throughput markers linked to the QTLs to enable marker assisted selection for the trait.

## Materials and methods

### Plant material

The GSB-resistant PI 482276 (*C. amarus*) (Gusmini et al. 2005) was crossed with susceptible Crimson Sweet (*C. lanatus*) in the greenhouse to generate an interspecific F_1_. A single F_1_ plant was self-pollinated to produce an F_2_ population. Individual F_2_ plants were self-pollinated to produce 178 F_2:3_ lines. Leaf material for parents, F_1_ and each F_2_ plant were collected and stored at   − 80 °C prior to DNA extraction.

### Inoculum preparation

A highly aggressive *Stagonosporopsis citrulli* isolate, 12178A (Gimode et al. 2019), kindly provided by Marin Brewer (University of Georgia, Department of Pathology), was grown (16 h/8 h light/dark cycle) on potato dextrose agar (PDA) (Becton, Dickinson and Company, NJ, USA) for 2 weeks. Approximately 1 cm^2^ agar plugs were then sub cultured on fresh PDA and grown for an additional 2 weeks. On the day of inoculation, PDA cultures were flooded with 10 ml of 0.1% tween20 and gently scraped with a microscope slide to release spores. The inoculum was filtered through 2 layers of sterile cheese cloth and spore concentration was determined using a hemacytometer (Hausser Scientific, PA, USA). Spore concentrations were adjusted to 5 × 10^5^ spores/ml using 0.1% tween20 solution.

### Phenotyping

The seeds of the F_2:3_ lines, F_1_, and parental lines were grown in the greenhouse in 48-cell seedling trays under LED lights (Fluence Science, TX, USA) until the 3–4 leaf stage (approximately 3 weeks). Seedlings were inoculated by spraying with 5 × 10^5^ spores/ml of freshly made *S. citrulli* inoculum until runoff. A randomized complete block design with three replicates was used, with four plants/genotype in each replicate (total of 12 F_3_ plants/genotype in each experiment). Seedlings were placed in a humidity chamber in the greenhouse for 3 days (avg ~ 23.5 °C and ~ 96% relative humidity) and then placed on a greenhouse bench and overhead watered as needed. Disease symptoms were scored 7 days post inoculation (dpi) at the whole plant level, for percentage of affected seedling using a 0–5 rating scale [(0 = no symptoms, 1 = 1 to 20%, 2 = 21 to 40%, 3 = 41 to 60%, 4 = 61 to 80% and 5 = more than 80% of the seedling covered with lesions) Electronic Supplementary Material 1]. Seedlings with a disease rating of 1 to 3 had lesions only on the leaves, while those with a disease rating of 4 and 5 had lesions on both leaves and stem. Two independent experiments were performed from Dec 16, 2019 to Jan 13, 2020 and from Jan 24 to Feb 24, 2020, respectively.

### Phenotypic data analysis

Analysis of variance (ANOVA) was performed using JMP® Pro 14.1 (SAS Institute Inc., Cary, NC) for the separate experiments with the effect of genotype and replication considered random. Further, an ANOVA of the combined experiments considered the random effects for genotype, replication, experiment and the interaction between genotype and experiment. BLUPs for each genotype, adjusted by the grand mean, was used for QTL mapping. Broad-sense heritability (H^2^) was calculated from the ANOVA table as V_g_ / [V_g_ + V_g×expt_/expt + V_e_/rep × expt], where *V*_g_ = genotypic variance, V_g×expt_ = genotype by experiment variance, *V*_e_ = error (residual) variance, rep = number of replications, and expt = number of experiments (Holland et al. 2003). The distributions of disease severity within each experiment and in the joined data were tested for deviations from normality with Shapiro–Wilk tests (Shapiro and Wilk 1965). Correlation between experiments was assessed using pairwise Pearson correlations (*r*) calculated in JMP® Pro 14.1 (SAS Institute Inc., Cary, NC).

### Genotyping, SNP analysis and map construction

For genotyping-by-sequencing (GBS) (Elshire et al. 2011), samples of the 178 F_2_ individuals, four PI 482276, four Crimson Sweet and four F_1_ individuals were freeze dried in two 96-well plates and shipped to Michigan State University for DNA extraction and quantification. Genomic DNA isolations and purifications were performed using the KingFisher flex (ThermoFisher Scientific Corporation, Waltham, MA) with Omega Mag-Bind kits (Omega Bio-Tek Inc., Norcross, GA). GBS was carried out at the Institute for Genomic Diversity, Cornell University, Ithaca, NY. Reads were aligned to the Charleston Gray watermelon genome (Wu et al. 2019) and SNPs were identified using TASSEL 5.0 GBS Discovery Pipeline (Glaubitz et al. 2014). The identified SNPs were filtered for polymorphism between the parents of the mapping population, missing data rate in the population (no more than 5%), and segregation distortion (*P* < 0.000001). A total of 1,525 SNPs were used for construction of the genetic map using the regression mapping method in JoinMap 5.0 (Van Ooijen 2006) and distances between markers were calculated using the Kosambi mapping function (Kosambi 1943).

### QTL mapping, candidate gene identification, and marker development

QTL detection for experiment 1, experiment 2 and the joined data was performed using composite interval mapping (CIM) (Zeng 1994) in WinQTLCart 2.5 (Wang et al. 2007). Threshold values for all traits were calculated through permutation tests (1,000 permutations, *α* = 0.05) (Churchill and Doerge 1994). CIM analysis was performed with a window size of 10 cM using the standard model (Model 6) with a walk speed of 1 cM and 5 marker cofactors determined by forward and backward regression.

The Charleston Gray (Wu et al. 2019) and the 97,103 v2 (Guo et al. 2019) watermelon genomes were used to identify candidate genes within the 2-LOD interval of significant QTLs. Syntenic regions associated with GSB resistance in other cucurbits were examined using the Synteny Viewer of the Cucurbit Genomics Database (Zheng et al. 2019) (https://cucurbitgenomics.org/). The genome resequencing data of PI 482276 (Guo et al. 2013) available at https://www.ncbi.nlm.nih.gov/sra/?term=SRP012850 was aligned to the Charleston Gray genome to identify polymorphisms in the candidate region. Pfam (https://pfam.xfam.org/) was used to determine protein domains likely to be associated with resistance.

KASP primers (Table 1) for SNPs closest to the QTL peaks were designed and optimized through Primer3Plus (Untergasser et al. 2007) and tested for polymorphisms with the two parents and the mapping population. The SNP of interest that was identified in the candidate gene region by aligning the genome resequencing reads of PI 482276 to the Charleston Gray reference genome was also developed into a KASP assay and the polymorphism was confirmed in the parents and the population. All KASP assays were carried out in 4-μl volumes containing 1.94 μl of 2 × low rox KASP master mix (LGC Genomics LLC, Teddington, UK), 0.06 μl primer mix with a final primer concentration of 0.81 μM, and 2 μl of 50–100 ng/μl genomic DNA. The PCR conditions used for the KASP assays consisted of an initial incubation at 95 °C for 15 min, 10 cycles of touchdown PCR with 20 s at 95 °C, 25 s of primer annealing temperature + 9 °C with 1 °C decrease each cycle, and 15 s of 72 °C, followed by 35 cycles of 10 s at 95 °C, 1 min at primer annealing temp, and 15 s at 72 °C, then held at 4 °C. KASP fluorescent end-point readings were measured using an Infinite M200Pro plate reader (Tecan Group Ltd.) and genotype calls were made using KlusterCaller (LGC Genomics LLC). Marker/trait association was analyzed using a one-way ANOVA followed by a Tukey–Kramer HSD test and R^2^ values (*P* = 0.05) determined in JMP® Pro 14.1 (SAS Institute Inc., Cary, NC).

**Table 1:** KASP™ assay primer sequences of SNPs associated with GSB resistance

KASP Assay	Ta (°C)	SNP	Primer type	Primer sequence (5′-3′)	Allele
ClGSB5.1–1	57	S05_33279166	FAM	GAAGGTGACCAAGTTCATGCTGACGAGGATGGTATGTTCAAATTCT	CS
			VIC	GAAGGTCGGAGTCAACGGATTGACGAGGATGGTATGTTCAAATTCG	PI 482276
			Reverse	GAACGAAGCAACCGCAATTC	
ClGSB7.1–1	58	S07_30544246	FAM	GAAGGTGACCAAGTTCATGCTCGGAGTCCGAAAGGATCTTCA	CS
			VIC	GAAGGTCGGAGTCAACGGATTCGGAGTCCGAAAGGATCTTCG	PI 482276
			Reverse	TTCCATGGCCTCTTCTGCAT	
ClGSB7.1–2	57.5	*ClCG07G013230;* 598 (C → T)	FAM	GAAGGTGACCAAGTTCATGCTTCCGCCGTCTTCTGCAAC	CS
			VIC	GAAGGTCGGAGTCAACGGATTTCCGCCGTCTTCTGCAAT	PI 482276
			Reverse	CCGATGCTGGTTAGGCAGTT	

## Results

### Phenotypic data

**Fig. 1:**
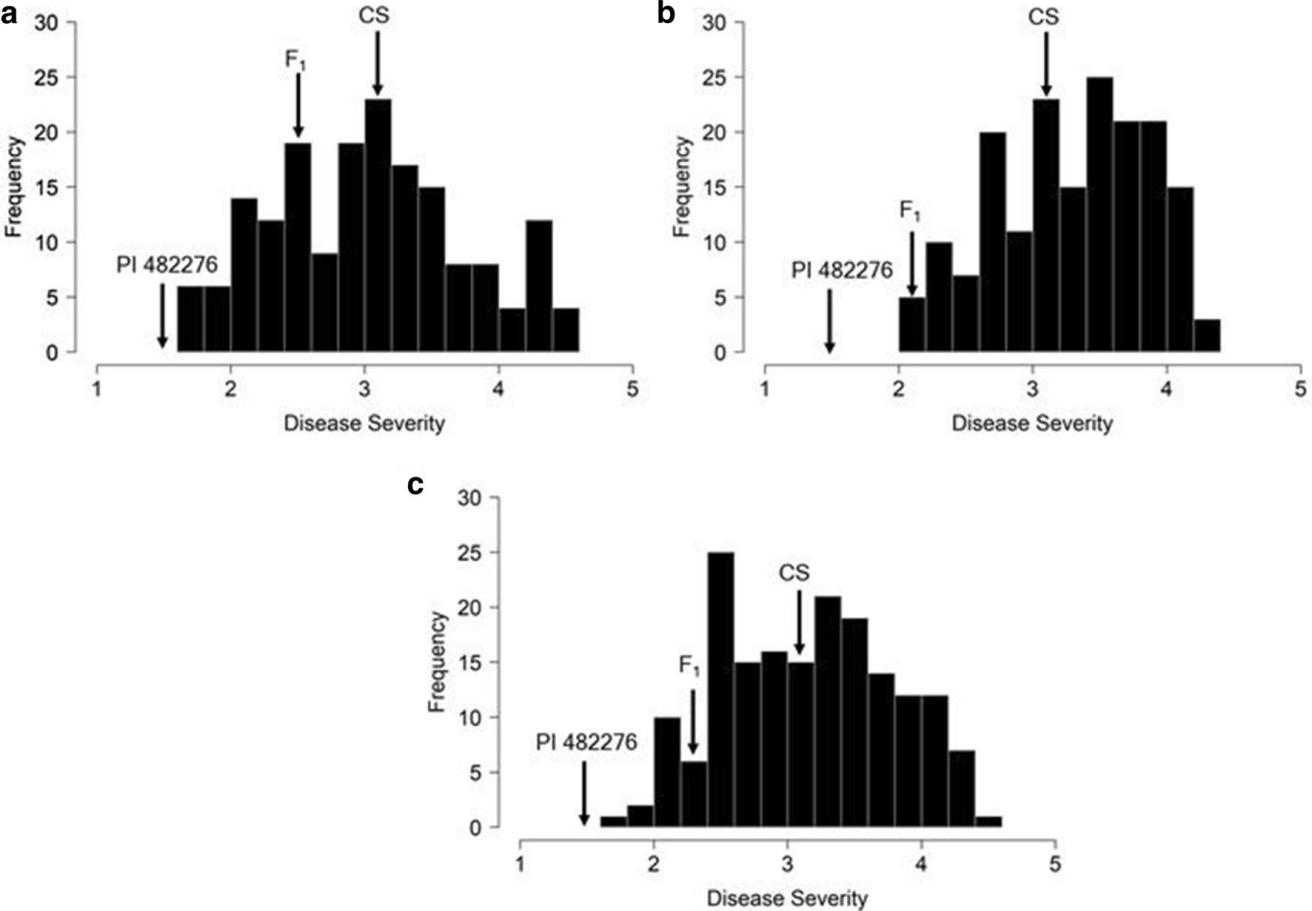
Frequency distribution for disease severity scores at 7 days post-inoculation with *Stagonosporopsis citrulli* for experiment 1 (**a**), experiment 2 (**b**) and joined data (**c**) in the Crimson Sweet (CS) × PI 482276 F_2:3_ watermelon population (*N* = 178)

The continuous phenotypic distributions of disease severity for the separate experiments as well as the joined data confirmed the quantitative nature of the trait (Fig. 1). All three distributions slightly deviated from a normal distribution according to the Shapiro–Wilk test for normality (*P* = 0.005, *P* = 0.001 and *P* = 0.008 for experiments 1, 2 and joined, respectively).

ANOVA showed significant effects for genotype (*P* < 0.0001) in individual (data not shown) and joined experiments (Table 2) but no significant effects were detected for the replication, experiment or interaction term of genotype × experiment in the joined analysis. The calculated variation between experiments accounted for only 0.7% of the total variation in GSB resistance, while replication and interaction of genotype by experiment contributed to 4.2% and 5.3%, respectively (Table 2). A significant (*P* < 0.0001) positive correlation (*r* = 0.57) was observed between the two experiments and the estimated broad sense heritability (H^2^) of resistance to GSB was 72.6% (Table 2).

**Table 2:** ANOVA table for variance components and broad sense heritability (H^2^) of gummy stem blight resistance in the Crimson Sweet × PI 482276 F_2:3_ watermelon population after inoculation with *Stagonosporopsis citrulli*

Variance component	Variance	Standard Error	%V_p_^a^
Genotype***	54.7	8.4	32.4
Experiment^NS^	1.1	5.5	0.7
Genotype × experiment^NS^	8.9	4.7	5.3
Replicate within experiment^NS^	7.1	5.4	4.2
Residual	97.1	5.2	57.5
H^2^	72.6		

^a^ Percent of total phenotypic variance

^***^Significant: *P* < 0.0001

^NS^: Not significant

### GBS, SNP analysis and map construction

A total of 36,797 SNPs were obtained from the GBS analysis and 10,112 were found to be polymorphic between Crimson Sweet and PI 482276. After filtering, a genetic map was created containing 1,525 SNP markers (Electronic Supplementary Material 2 and 3) with a 1.2 cM average distance between markers and a total length of 1,744 cM. Two regions on chromosome 8 and one on chromosome 5 had large gaps between markers: 25.58 cM and 21.71 cM on chromosome 8, and 18.77 cM on chromosome 5 (Electronic Supplementary material 2).

### QTL identification

**Fig. 2:**
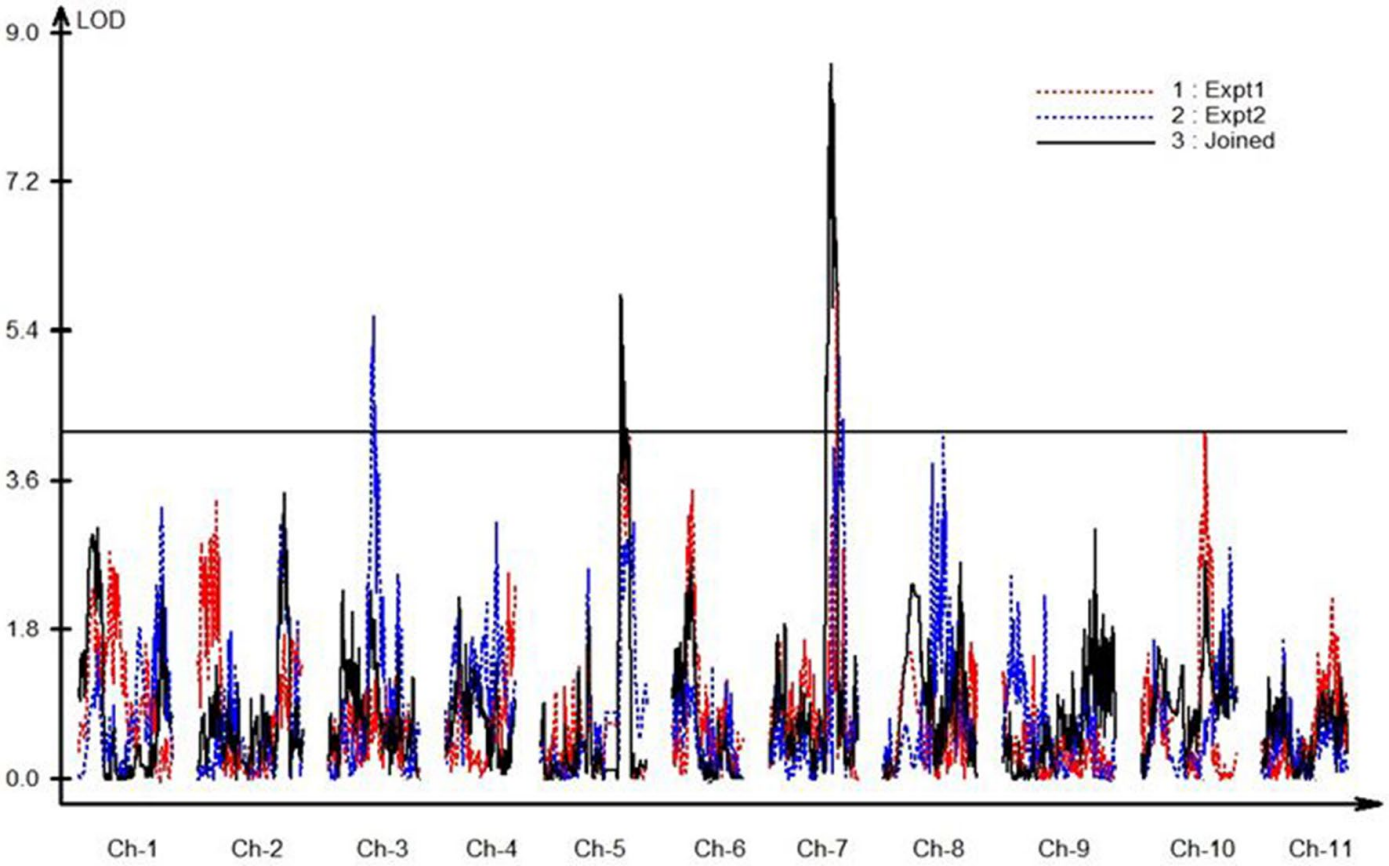
QTL associated with GSB resistance in the Crimson Sweet (CS) × PI 482276 F_2:3_ watermelon population (*N* = 178) in experiment 1, experiment 2 and joined data

GSB QTLs were identified on chromosomes 3, 5 and 7. In experiment 1, QTLs were identified on chromosomes 5 (*ClGSB5.1*: R^2^ = 6.4%; 135.3–145.3 cM) and 7 (*ClGSB7.1*: R^2^ = 15.4%; 114.3–116.3 cM) with maximum LOD scores of 4.4 and 6.5, respectively. In experiment 2, QTLs were identified on chromosomes 3 (*ClGSB3.1*: R^2^ = 14.1%; 76–79.1 cM) and 7 (*ClGSB7.1*: R^2^ = 16%; 117.7–129 cM), with maximum LOD scores of 5.6 and 5.1, respectively. For the joined analysis, QTLs with LOD scores of 5.9 and 8.6 were identified on chromosomes 5 (135.3–141.2 cM) and 7 (103.1–116.3 cM), explaining 10.2% and 21.1% of the phenotypic variance, respectively. These two QTLs of the joined analysis both co-localized with the QTLs for experiment 1 (Table 3 and Fig. 2). The closest SNP to the QTL peaks for *ClGSB3.1, ClGSB5.1* and *ClGSB7.1* were S03_12292063 (76.83 cM; experiment 2), S05_33279166 (139.22 cM; joined) and S07_30544246 (106.35 cM; joined), respectively.

**Table 3:** Quantitative trait loci (QTL) associated with resistance to *Stagonosporopsis citrulli* and the corresponding 2-LOD support interval for separate and joined data for disease severity observed at 7 d post-inoculation from two screens of the Crimson Sweet × PI 482276 F_2:3_ watermelon population

Trait	QTL name	Chromosome	Peak (cM)	LOD^a^	Additive^b^	Dominance^c^	2- LOD interval (cM)^d^	Right flanking marker (Mb)	Left flanking marker (Mb)	R^2^(%)^e^
Expt 1	*ClGSB5.1*	5	143.41	4.4	0.2851	0.0385	135.3–145.3	24,766,876	33,886,603	6.4
Expt 1	*ClGSB7.1*	7	114.71	6.5	0.3655	− 0.0876	114.3–116.3	31,234,486	31,729,927	15.4
Expt 2	*ClGSB3.1*	3	76.81	5.6	− 0.1394	0.3044	76.0–79.1	12,183,997	16,245,609	14.1
Expt 2	*ClGSB7.1*	7	119.11	5.1	0.2496	− 0.169	117.7–129.0	31,760,991	32,678,198	16.0
Joined	*ClGSB5.1*	5	137.71	5.9	0.3354	0.0918	135.3–147.1	24,766,876	33,964,634	10.2
Joined	*ClGSB7.1*	7	105.61	8.6	0.3517	− 0.0996	103.1–116.3	25,992,663	31,729,927	21.1

^a^Logarithm of odds ratios at the position of the peak

^b^Additive effect of QTL

^c^Dominance effect of QTL

^d^The QTL interval on genetic map

^e^Percent of phenotypic variance explained by the QTL

### Candidate gene identification

The total number of genes in the 2-LOD confidence interval for each QTL were: *ClGSB3.1:* 65; *ClGSB5.1:* 712; *ClGSB7.1:* 574 (Electronic Supplementary Material 4). The GSB resistance loci and candidate genes identified in the present study were compared with those identified previously in cucurbit species (Lou et al. 2013; Liu et al. 2017; Zhang et al. 2017; Hassan et al. 2018; Hu et al. 2018; Ren et al. 2019).

*ClGSB7.1* was responsible for the highest phenotypic variance and harbors several disease resistance-related genes. Among them are four nucleotide-binding site leucine-rich repeat (NBS-LRR) genes (*ClCG07G015790, ClCG07G015810, ClCG07G015870* and *ClCG07G015880*), and those encoding LRR containing proteins (*ClCG07G010720, ClCG07G012370, ClCG07G013540, ClCG07G014060, ClCG07G014730, ClCG07G015010, ClCG07G015800, ClCG07G015890*), receptor-like protein kinases (RLK), including LRR-RLKs (*ClCG07G010330, ClCG07G011290, ClCG07G011830, ClCG07G011880, ClCG07G012440, ClCG07G013510, ClCG07G014170, ClCG07G014760, ClCG07G015750*) and an Avr9/Cf-9 rapidly elicited disease resistance protein (*ClCG07G013230*) (Electronic Supplementary Material 4).

**Fig. 3:**
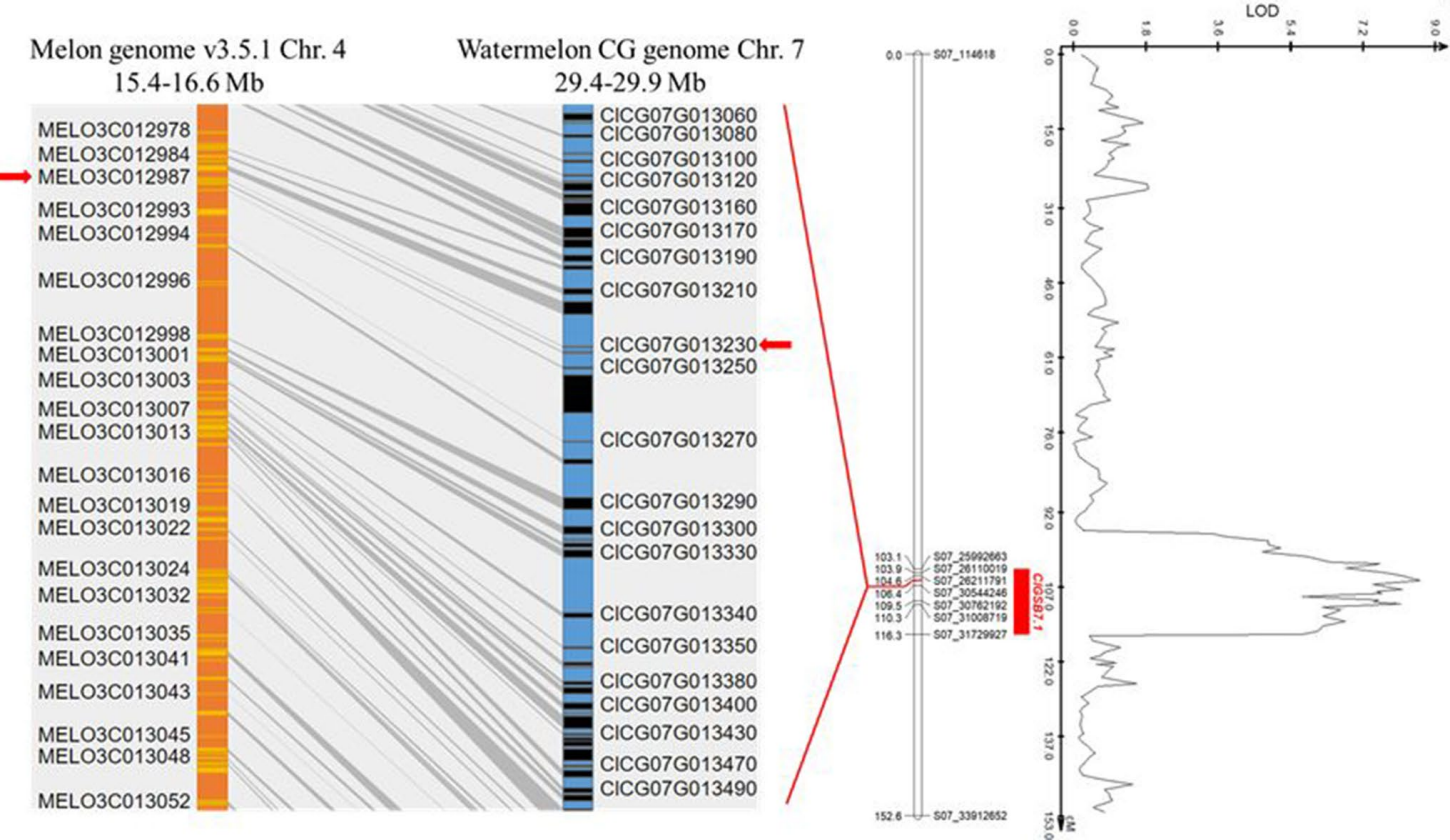
Syntenic analysis of the GSB resistance region in *Cucumis melo* (Hu et al. 2018) with the QTL region on *Citrullus lanatus* chromosome 7. Orange and blue represent *Cucumis melo* and *Citrullus lanatus* chromosome 4 and 7, respectively. The disease resistance melon gene *MELO3C012987* is an ortholog of watermelon gene *ClCG07G013230*

Syntenic analysis revealed conserved synteny between *ClGSB7.1* and a locus in *Cucumis melo* (melon) chromosome 4 associated with GSB resistance (Hu et al. 2018) (Fig. 3). Eight candidate genes were reported in a 0.667 cM QTL region of chromosome 4 (Hu et al. 2018) and *MELO3C012987,* which displayed differential expression and sequence polymorphism between the resistant and susceptible melon lines, was determined as the most likely candidate gene associated with GSB resistance (Hu et al. 2018). In watermelon, *ClCG07G013230* is an ortholog of *MELO3C012987.*

**Fig. 4:**
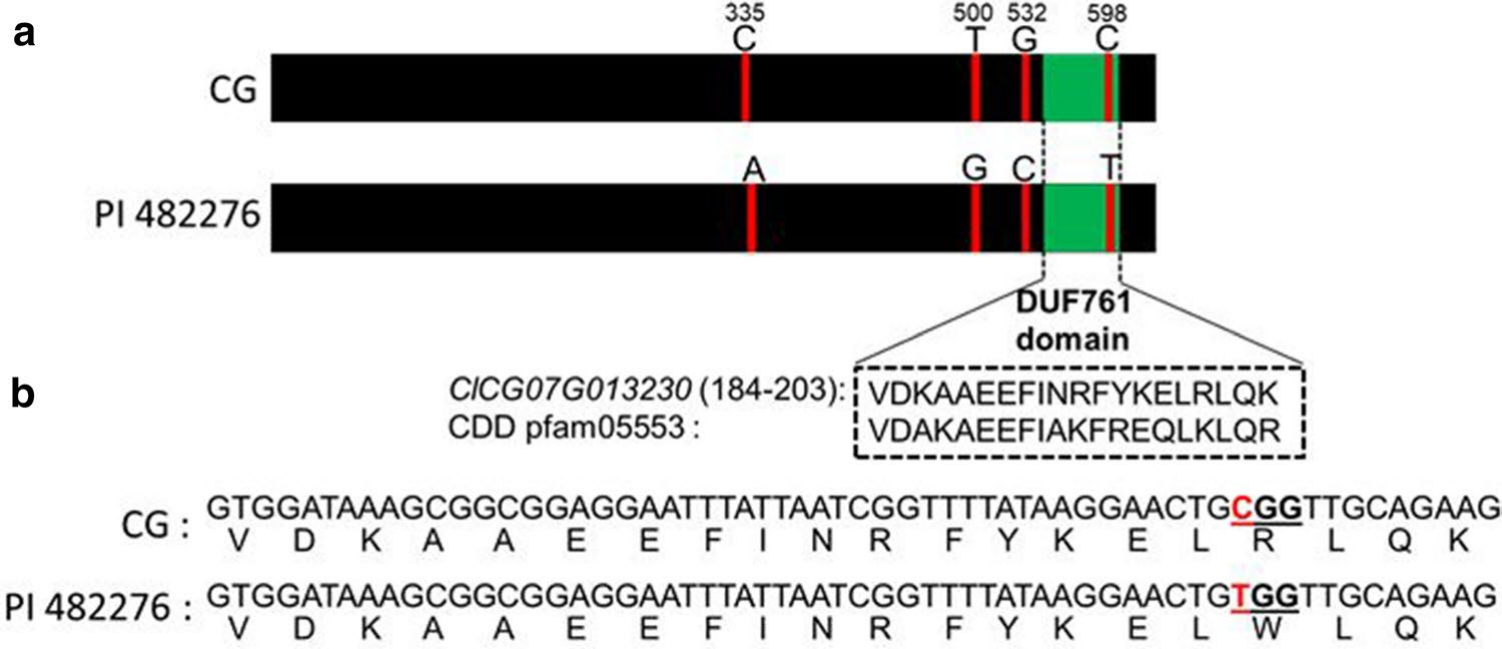
Alignment of the *ClCG07G013230* gene between the Charleston Gray (CG) reference genome and PI 482276. **a** SNPs and their bp positions on the gene. **b** The DUF761 domain with a non-synonymous substitution from C to T that changes the amino acid from Arginine to Tryptophan

The *ClCG07G013230* (29,622,088—29,622,708 Mb) gene is 621 base pairs (bp) long and contains no introns. Alignment of the PI 482276 sequence (Guo et al. 2013) to the Charleston Gray genome (Wu et al. 2019) revealed four SNPs in the gene between the two genotypes. The four point mutations at bp positions 335 (C → A), 500 (T → G), 532 (G → C) and 598 (C → T) (Fig. 4) were all non-synonymous and the base substitutions cause an amino acid change from Alanine to Glutamic acid, Valine to Glycine, Alanine to Proline and Arginine to Tryptophan, respectively. A Pfam domain analysis indicated that *ClCG07G013230* harbors a DUF761 domain between 550 and 609 bp of the gene, which includes the Arginine to Tryptophan amino acid change. KASP assay confirmed the presence of the 598 (C → T) polymorphism between Crimson Sweet and PI 482276.

The candidate genes underlying *ClGSB5.1* include those encoding F-box family proteins with LRR domains (*ClCG05G015740, ClCG05G015980, ClCG05G016910, ClCG05G020150*, *ClCG05G020210, ClCG05G020550*), RLKs (*ClCG05G014900, ClCG05G015780, ClCG05G017510, ClCG05G017520, ClCG05G018400, ClCG05G018970*), an enhanced disease resistance 2-like lipid binding protein (EDR2; *ClCG05G016060*), a non-race specific disease resistance protein (*ClCG05G014750*) and one NBS-LRR gene (*ClCG05G019540*). *ClCG05G019540* is equivalent to the *Cla020705* in the 97103_V1 genome (Guo et al. 2013) and encodes proteins with both RPW8 and NBS-LRR domains. *Cla020705* was differentially expressed between resistant PI 189225 and susceptible Charleston Gray inoculated with *D. bryoniae* (Hassan et al. 2019). *ClCG05G019540* (31,779,884—31,783,097 Mb) is 3,214 bp long and contains five exons. Thirteen SNPs were identified on the coding sequence of *ClCG05G019540* between PI 482276 and Charleston Gray but all of them were synonymous. Among the 65 genes on *ClGSB3.1*, one of the genes is an LRR-RLK (*ClCG03G009870*) (Electronic Supplementary Material 4).

### Marker performance

**Fig. 5:**
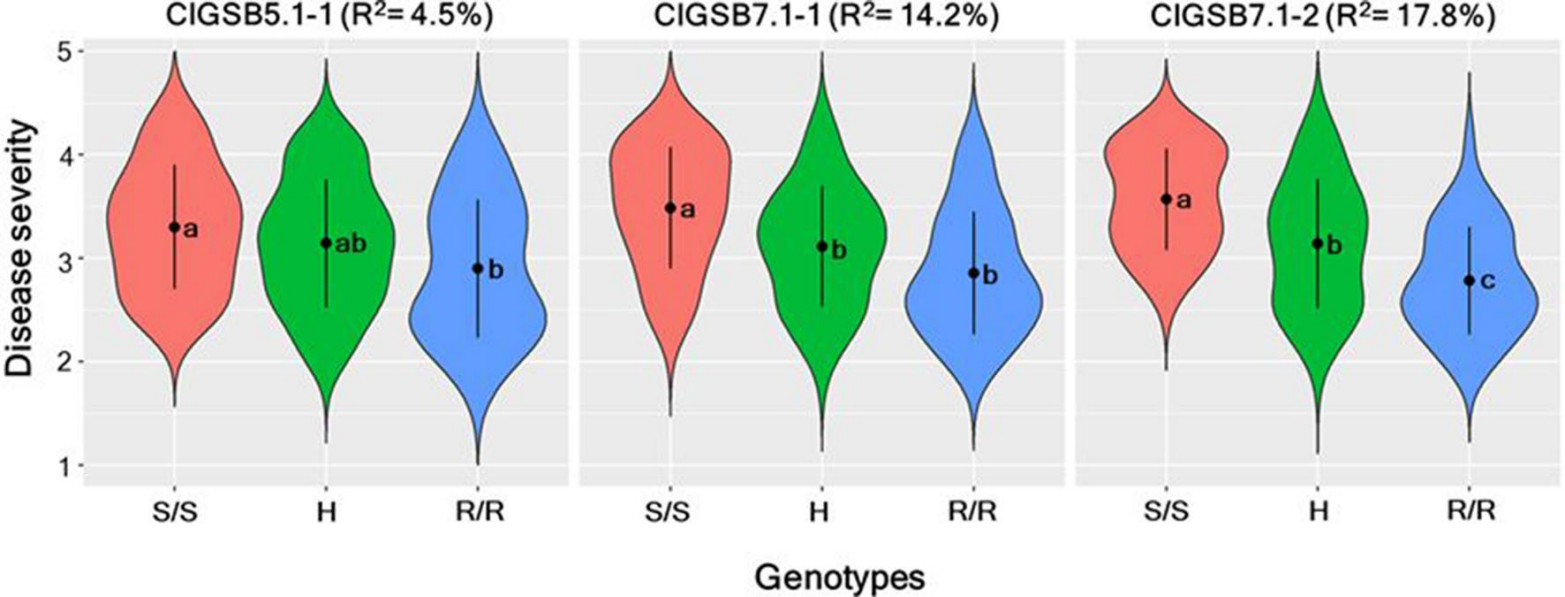
Performance of KASP assays in the Crimson Sweet (CS) × PI 482276 F_2:3_ mapping population. Dots indicate means and levels not connected by the same letter are significantly different, where S represents the susceptible (CS) allele, H represents the heterozygote and R represents the resistant (PI 482276) allele

High throughput KASP assays were developed for the SNPs closest to the peaks of *ClGSB5.1* and *ClGSB7.1* (Table 1). For all assays, disease severity was significantly lower for individuals homozygous for the resistant allele (R/R) than individuals homozygous for the susceptible allele (S/S) (Fig. 5). Assay ClGSB5.1–1 (S05_33279166; 139.22 cM) showed a significant (*P* = 0.019) association with disease severity (RR = 2.9; SS = 3.3) and had an R^2^ value of 4.5%. This marker had significant segregation distortion (*P* = 0.01), with the homozygous resistant genotype being underrepresented. Similar segregation distortion was observed for all the markers in this QTL region.

The closest marker to the QTL peak for *ClGSB7.1* (105.61 cM) was S07_30544246 (106.35 cM), and associated assay, ClGSB7.1–1, showed a significant difference (*P* < 0.0001) in disease severity between progeny homozygous for the PI 482276 (RR = 2.86) and the Crimson Sweet alleles (SS = 3.49) in the population (R^2^ = 14.2%).

ClGSB7.1–2, the KASP assay designed for the non-synonymous SNP in the DUF761 domain of *ClCG07G013230* gene, was polymorphic between the two parents (CS and PI 482276). This SNP mapped at 105.08 cM (data not shown), between S07_26211791 and S07_30544246 and displayed the highest R^2^ value of 17.8% in the mapping population. Progeny with the 3 different ClGSB7.1–2 genotypes were significantly different from each other [(*P* < 0.0001) (Fig. 5)] and the mean disease severity scores for progeny with the RR and SS genotypes were 2.79 and 3.57, respectively.

## Discussion

The phenotypic distribution in the F_2:3_ population from the Crimson Sweet × PI 482276 cross was continuous, confirming the quantitative nature of GSB resistance in this population. Transgressive segregation was observed in the direction of susceptibility. The correlation between the two experiments performed was moderate (*r* = 0.57). Variability in evaluations for GSB resistance in cucurbits has been observed in previous studies. A study by Wehner and Shetty (2000) reported low correlation between GSB ratings in cucumber, while Zhang et al. (1997) reported significantly high correlations (*r* = 0.50–0.92) among greenhouse experiments and inoculated field trials. Gusmini et al. (2005) reported a low correlation (*r* = 0.10 – 0.36) in the evaluation for GSB resistance in watermelon. More recently, Ren et al. (2019) reported a significantly high correlation (*r* = 0.92) for GSB disease incidence in watermelon seedlings between two greenhouse experiments. We calculated a relatively high (72.6%) broad sense heritability, with no significant interaction observed between the genotype × experiment. Previous QTL studies on GSB resistance (Lou et al. 2013; Liu et al. 2017; Zhang et al. 2017; Ren et al. 2019) did not partition the overall variance into genetic versus environmental components so it was not possible to compare the heritability estimates with other findings. Due to the non-significant experiment, and genotype-by-experiment interaction, the joined analysis data was considered most informative as it incorporated data from 24 plants/genotype, which gives a more accurate estimate of the F_2:3_ family means.

Different modes of inheritance have been proposed for GSB resistance in cucurbits, including monogenic recessive (Norton 1979; Frantz and Jahn 2004; Hassan et al. 2018), monogenic dominant (Zuniga et al. 1999; Frantz and Jahn 2004; Wolukau et al. 2007; Hu et al. 2018), and polygenic (Lou et al. 2013; Gusmini et al. 2017; Liu et al. 2017; Zhang et al. 2017; Hassan et al. 2019; Ren et al. 2019) inheritance patterns. QTLs associated with GSB resistance have been described in cucumber (Lou et al. 2013; Liu et al. 2017; Zhang et al. 2017) and watermelon (Ren et al. 2019). In cucumber (*C sativus* L.), the study by Lou et al. (2013) used introgression lines to identify two QTLs on chromosomes 4 (GSB4) and 6 (GSB6b), spanning 12 cM and 11 cM, respectively. Liu et al. (2017) utilized recombinant inbred lines (RILs) to identify six QTLs associated with GSB resistance in cucumber seedlings, of which one (*gsb5.1*) was stable for three seasons and explained 17.9% of the phenotypic variation. One hundred and two candidate genes were predicted in the 0.5 cM QTL region, and seven genes related to disease resistance were identified (Liu et al. 2017). Five QTLs conferring GSB resistance in the cucumber stem were identified by Zhang et al. (2017) in a RIL population and the locus on chromosome 6 (*gsb-s6.2*) accounted for the highest phenotypic variation of 22.7%. One hundred and seventeen candidate genes were predicted in the 3.2 cM QTL region, of which fourteen were related to disease resistance (Zhang et al. 2017). In melon (*Cucumis melo* L.), GSB resistance QTLs have not been described, however, Hassan et al. (2018) aligned known GSB QTL segments from the cucumber genome with the melon genome to discover genes associated with GSB resistance. A QTL associated with GSB resistance in watermelon was recently mapped on chromosome 8 (*Qgsb8.1*) using PI 189225 as the resistance source (Ren et al. 2019). *Qgsb8.1* spans a 571.27 kb region and contains approximately nineteen annotated genes, two of which are related to disease resistance. We identified three QTLs significantly associated with GSB resistance on chromosomes 3, 5 and 7 of watermelon in an interspecific Crimson Sweet × PI 482276 cross, which represent a novel source of resistance to GSB. It is worth noting that the location of *Qgsb8.1* described by Ren et al. (2019) is within the 21.71 cM (10.2 Mbp) gap on chromosome 8 on our genetic map. Due to low marker density in this region, we cannot determine the potential association of the region with GSB resistance in the present study. The large genetic distances between markers in this location may be due differences in chromosome structure and distorted segregation that often occurs in interspecific crosses. Sandlin et al. (2012) reported a 33.04 cM gap between markers in the ZWRM50 (*C. lanatus*) × PI 244019 (*C. amarus*) map. Another possibility for lack of detection of *Qgsb8.1* in our population could be the utilization of different resistance sources and pathogen isolates in the two studies. Ren et al. (2019) used PI 189225 and a *S. cucurbitacearum* isolate while we used PI 482276 and a *S. citrulli* isolate. It is still unclear whether the resistance loci provide resistance across different *Stagonosporopsis* species. Additional fine mapping of the three loci identified in this study will be needed to better understand resistance to GSB in PI 482276.

*ClGSB7.1* was stable across the two experiments while *ClGSB5.1* and *ClGSB3.1* were dependent on the environment. *ClGSB7.1* appears to have the greatest potential for introgression into cultivated watermelon since it not only explained the highest proportion of variation in GSB resistance (21%) but was also stable in the two experiments and the joined analysis. None of the progeny in our study was as resistant as the PI 482276 parent (Fig. 1), however selecting for *ClGSB7.1* provides an intermediate level of resistance (disease severity = 2.79). Further research is needed to determine the effectiveness of this level of resistance under field conditions. Efforts to breed for resistance to GSB in watermelon began in the 1970s (Norton 1979; Norton et al. 1986) but to date, no commercial cultivars with field-level resistance have been developed. It is likely that this is at least partially due to the complex genetic control of GSB resistance by separate loci with different effects. The identification of several resistance QTLs from different resistance sources would allow for pyramiding multiple resistance alleles into cultivated watermelon.

The significant loci detected in this study all harbor potential candidate genes including those encoding NBS-LRRs, LRR domains, RLKs, an Avr9/Cf-9 protein, an EDR2 protein and a non-race specific disease resistance protein, which are all associated with plant defense against pathogens. Further fine mapping will be needed to narrow down the number of candidate genes in the QTLs. An examination of published research on gummy stem blight resistance in cucurbits did however reveal candidate genes identified by previous research within the regions of interest of the current study. The NBS-LRR *ClCG05G019540* gene found in *ClGSB5.1* had thirteen synonymous SNPs in the exons. Hassan et al. (2019) found that *Cla020705* (*ClCG05G019540*) exhibited higher expression in resistant (PI 189225) compared to the susceptible (Charleston Gray) watermelon, which could be due to mutations in the promoter region of this gene. Further functional analysis through expression studies of this gene between PI 482276 and Crimson Sweet may provide a better understanding of the association of *ClCG05G019540* with GSB resistance in this genetic background. The four SNPs found in the *ClCG07G013230* gene in *ClGSB7.1* all led to a change in amino acid. ClGSB7.1–2 KASP assay for the C → T SNP in the DUF761 domain which is associated with disease resistance (Zhang et al. 2019), was polymorphic between Crimson Sweet and PI 482276, confirming what was observed from the genome alignments. This SNP displayed significant association with GSB resistance in the mapping population. We therefore propose *ClCG07G013230* as a candidate gene for resistance to GSB in watermelon. However, additional research will be required to fine map the region to exclude other potential candidate genes. *ClCG07G013230* should also be sequenced in the parental lines to confirm the sequence information from the mined data. Future research will include gene expression studies comparing PI 482276 and susceptible watermelon lines to confirm its role in GSB resistance. The utility of the KASP assays described in this study needs to be validated in other genetic backgrounds to confirm their usefulness in marker-assisted selection (MAS) for GSB resistance in watermelon breeding.

One of the major drawbacks in the quest to breed for GSB-resistant cultivars has been the labor-intensive phenotyping process and inconsistencies observed with phenotyping results (Wehner and Shetty 2000; Gusmini et al. 2005; Wehner 2008). Application of molecular breeding tools such as marker-assisted selection would greatly improve GSB-resistant cultivar development by minimizing the labor-intensive and time-consuming steps in the breeding process. We have developed high throughput KASP assays for MAS that will allow for more efficient incorporation of GSB resistance into elite watermelon cultivars.

## Electronic supplementary material

Below is the link to the electronic supplementary material.

Supplementary file1 (DOCX 2140 kb)

Supplementary file2 (DOCX 127 kb)

Supplementary file3 (XLSX 975 kb)

Supplementary file4 (XLSX 81 kb)

## Data Availability

Data is provided in Electronic Supplementary Material.
