# Supplementary material for: QTL associated with gummy stem blight resistance in watermelon
Source: Theor Appl Genet. 2020 Nov 1;134(2):573–84. doi: 10.1007/s00122-020-03715-9 (PMC7843542; doi:10.1007/s00122-020-03715-9)
Supplement: Supplementary file 1 — Supplementary file1 (DOCX 2140 kb) [file 122_2020_3715_MOESM1_ESM.docx]

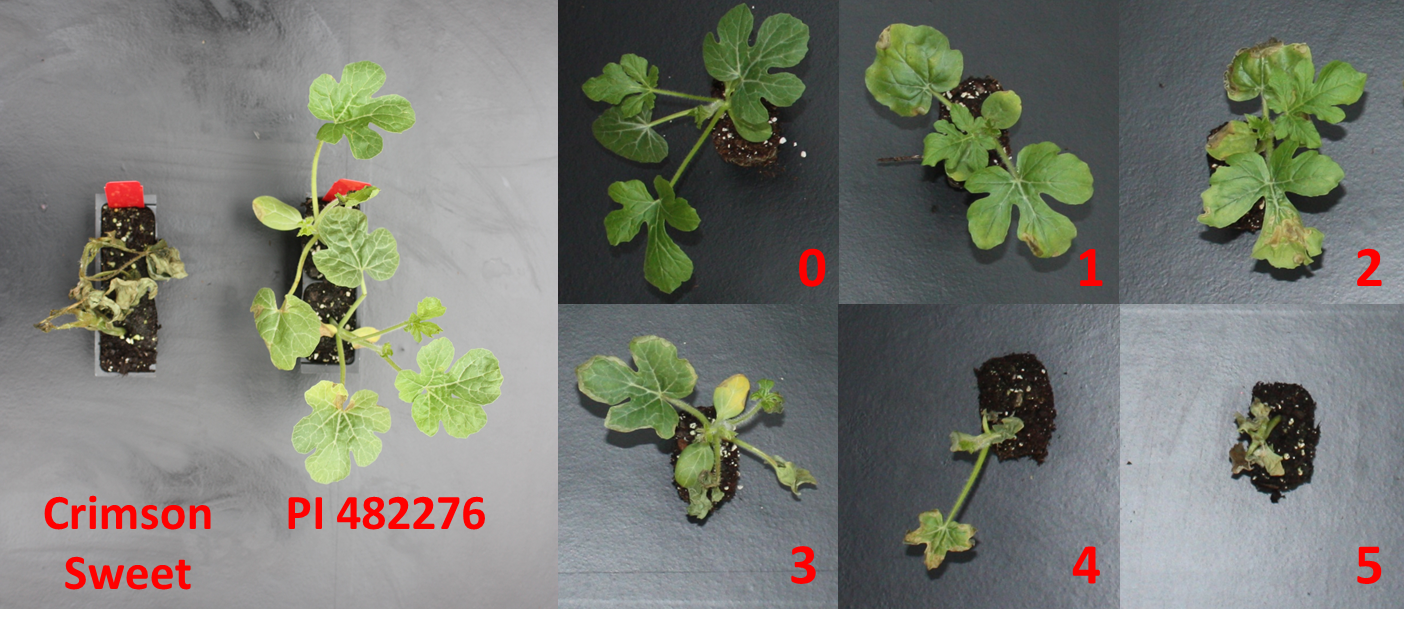


Electronic Supplementary Material 1: Parental phenotypes and the 0-5 disease rating scale of seedlings inoculated with *Stagonosporopsis citrulli* 12178A.
