# Supplementary material for: QTL associated with gummy stem blight resistance in watermelon
Source: Theor Appl Genet. 2020 Nov 1;134(2):573–84. doi: 10.1007/s00122-020-03715-9 (PMC7843542; doi:10.1007/s00122-020-03715-9)
Supplement: Supplementary file 2 — Supplementary file2 (DOCX 127 kb) [file 122_2020_3715_MOESM2_ESM.docx]

Electronic Supplementary Material 2: Crimson Sweet (*Citrullus lanatus*) × PI 482276 (*Citrullus amarus*) genetic linkage map with 1,525 SNPs.
